# Loss-of-function manipulations to identify roles of diverse glia and stromal cells during CNS scar formation

**DOI:** 10.1007/s00441-021-03487-8

**Published:** 2021-06-24

**Authors:** Shalaka Wahane, Michael V. Sofroniew

**Affiliations:** Department of Neurobiology, David Geffen School of Medicine, University of California, Los Angeles, CA 90095 USA

**Keywords:** CNS scar formation, Diverse glia and stromal cells, Loss-of-function manipulations

## Abstract

Scar formation is the replacement of parenchymal cells by stromal cells and fibrotic extracellular matrix. Until as recently as 25 years ago, little was known about the major functional contributions of different neural and non-neural cell types in the formation of scar tissue and tissue fibrosis in the CNS. Concepts about CNS scar formation are evolving rapidly with the availability of different types of loss-of-function technologies that allow mechanistic probing of cellular and molecular functions in models of CNS disorders in vivo. Such loss-of-function studies are beginning to reveal that scar formation and tissue fibrosis in the CNS involves complex interactions amongst multiple types of CNS glia and non-neural stromal cells. For example, attenuating functions of the CNS resident glial cells, astrocytes or microglia, can disrupt the formation of limitans borders that form around stromal cell scars, which leads to increased spread of inflammation, increased loss of neural tissue, and increased fibrosis. Insights are being gained into specific neuropathological mechanisms whereby specific dysfunctions of different types of CNS glia could cause or contribute to disorder-related tissue pathology and dysfunction. CNS glia, as well as fibrosis-producing stromal cells, are emerging as potential major contributors to diverse CNS disorders either through loss- or gain-of-functions, and are thereby emerging as important potential targets for interventions. In this article, we will review and discuss the effects on CNS scar formation and tissue repair of loss-of-function studies targeted at different specific cell types in various disorder models in vivo.

## Introduction: lesions, inflammation, and scar formation in the CNS

All organs consist of parenchymal cells (also known as principle cells) that carry out the specific functions of the organ and stromal cells (fibroblast lineage cells, pericytes, vasculature, etc.) that form connective tissue and provide support (Rhett et al. 2008; Rog-Zielinska et al. 2016). Scar formation is the replacement of parenchymal cells by stromal cells and fibrotic extracellular matrix, which can occur in all organs throughout the body including the central nervous stem (CNS) during injury or disease. In the CNS, scar formation can occur in response to acute or chronic insults, including trauma, ischemia, microbial infection, seizures, autoimmune inflammation, tumours, environmental toxins, peripheral metabolic disorders, or neurodegenerative disease. Scar formation and tissue fibrosis can occur in the CNS over a wide range of scales from very large in lesions caused by trauma or stoke to very small in response to secondary inflammation that accompanies multiple disorders. Consequently, there is much interest in understanding and modulating CNS scar formation and tissue fibrosis in order to identify ways to improve CNS repair and functional outcome across a wide spectrum of disorders.

Concepts about CNS scar formation are evolving rapidly with the availability of many new technologies that allow mechanistic probing of cellular and molecular functions in models of CNS disorders in vivo. Until as recently as 25 years ago, little was known about the major functional contributions of different neural and non-neural cell types in the formation of scar tissue and tissue fibrosis. This is now an area of intense investigation. It has become clear that CNS responses across the spectrum insults are multicellular and intensely interactive, with complex inter-dependent interactions amongst diverse neural and non-neural cells (Burda and Sofroniew 2014). The roles of different cell types are gradually being characterised. Notably, glial cells, long regarded as forming purely detrimental ‘glial scars’, are emerging as CNS parenchymal cells that perform essential neural repair functions that constrain true scar formation and fibrosis by non-neural cells. Indeed, the terminology that refers to glia as ‘scars’ is being challenged, and it can be argued that glia are CNS parenchymal cells that promote tissue repair by replacing lost neural parenchyma with new neural parenchyma rather than with fibrotic tissue.

One of the major ways to dissect and understand cellular mechanisms is with loss-of-function manipulations that target the ablation of specific cell types or target the deletion of specific molecules from specific cells. In this article, we will review the effects on CNS scar formation and tissue repair of loss-of-function studies targeted at different specific cell types in various disorder models in vivo. We will discuss the organisation of CNS lesions and the evolving concept of what does, and what does not, comprise scar tissue in the CNS. We will consider how scar formation relates to CNS mechanisms that exist to attract and control immune responses that are essential for clearing microbial infections. We will examine how loss- or gain-of-function studies not only foster mechanistic understanding, but also provide insight into the consequences of manipulations intended to modulate scar formation and thereby inform the development of beneficial therapeutic approaches.

## Multicellular organisation of CNS lesions with scars

**Fig. 1:**
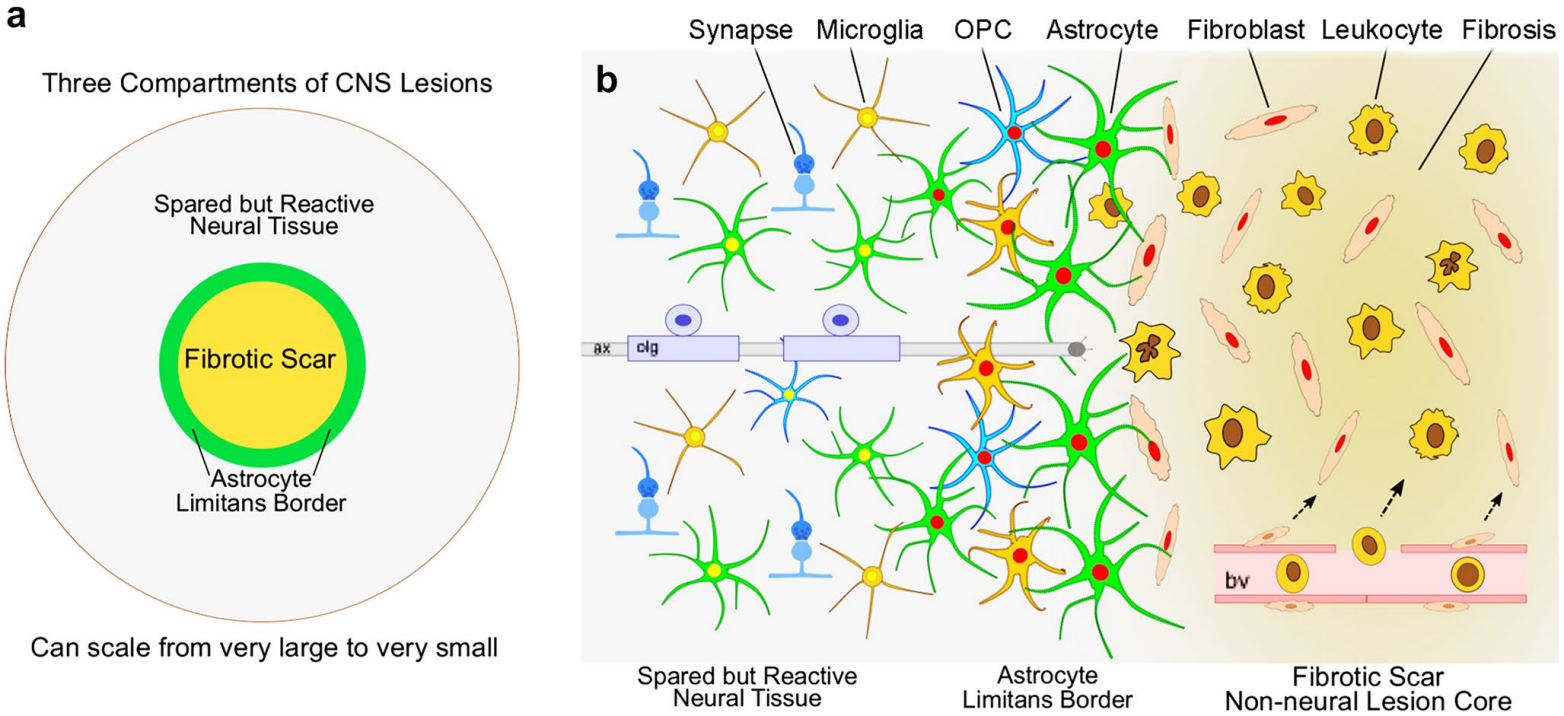
Schematic illustrations showing the overall spatial organization **a** and the main cellular components **b** of the three concentric tissue compartments that comprise CNS lesions: (i) fibrotic scar as the non-neural lesion core, (ii) astrocyte limitans border, and (iii) spared but reactive neural tissue. (i) Non-neural lesion cores form in response to the death and degeneration of CNS neural parenchyma. Microglia and astrocytes recruit professional blood-borne immune and inflammatory cells to assist with debris clearance and monitor for microbial infection. Simultaneously, perivascular fibroblasts and pericytes proliferate (red nuclei in **b**) and migrate to repopulate areas of lost parenchyma with scar forming fibroblasts that produce fibrotic extracellular matrix. (ii) Astrocyte limitans borders form through the interactions of astrocytes, microglia, and OPCs, which proliferate (red nuclei in **b**) and migrate to surround and restrict the migration of stromal and inflammatory cells. Astrocyte limitans borders adjacent viable neural tissue by corralling scar forming fibroblasts and inflammatory cells restricting their migration. Like astrocyte limitans borders along meninges, astrocyte limitans borders around stromal cell scars are narrow and only several cell layers thick, even when stromal cell scars are very large. (iii) Spared but reactive neural tissue is immediately adjacent to and directly continuous with astrocyte limitans borders and is characterised by the presence of glia that are reactive but generally not proliferative, including astrocytes, microglia, and OPCs. This spared but reactive neural tissue is undergoing synapse turnover and circuit reorganization. *ax* axon, *bv* blood vessel, *olg* oligodendrocyte

To productively study the effects of loss-of-function manipulations on scar formation in the CNS, it is important first to understand the multicellular organisation of mature CNS lesions that contain scar tissue and the different phases of scar formation. In the CNS, as in other organs, scar formation can be parsed broadly into three overlapping but distinct phases: (i) degeneration and death of CNS parenchymal cells resulting in inflammation, (ii) cell proliferation and tissue replacement, and (iii) tissue remodelling as reviewed in detail elsewhere (Burda and Sofroniew 2014) and briefly summarised here. (i) Insults that cause neural cell death or degeneration can range in scale from the very large to very small and can be acute or chronic, as for example in acute and very large strokes or traumatic brain or spinal cord injuries (TBI or SCI), or in small chronic insults resulting from repetitive microinfarcts, autoimmune infiltration, or degenerative disease. In response to cell damage and degeneration, molecular cues are induced that attract immune and inflammatory cells to clear debris and that drive proliferative responses of surrounding neural parenchymal glial cells (Burda and Sofroniew 2014). (ii) In most CNS areas, lost neurons cannot be replaced and regions devoid of neuronal elements (neuronal cell bodies or axons) become occupied by proliferating stromal cell scar tissue. In parallel, multiple glial cell types proliferate around the margins of lost neural tissue and interact to form an astrocyte limitans border that delineates viable neural tissue from stromal cell scar tissue. These borders appear similar in form and function to the astrocyte limitans borders that separate neural tissue from meningeal stromal cells around the entire CNS (Sofroniew 2015). The formation of these limitans borders involves proliferation and interactions amongst various CNS resident glial cells, astrocytes, and microglia, with non-neural stromal cells as described in more detail in the sections on these cell types below. (iii) The tissue remodelling phase can occur over a prolonged period during which stromal cells produce extracellular matrix resulting in fibrotic scars. After acute single injuries, stromal scars and their astrocyte borders contract but persist. In the presence of chronic insults, the replacement of neural tissue with stromal cells and fibrosis can gradually spread. In either case, immediately adjacent to scars and their narrow astrocyte limitans borders, there are directly continuous areas of reactive but functional neural tissue undergo synapse remodelling and circuit reorganisation. These events have been reviewed in more detail elsewhere (Burda and Sofroniew 2014; O’Shea et al. 2017; Sofroniew 2015, 2020). As a result of the multicellular interactions that give rise to scar formation and tissue fibrosis, mature lesions in the CNS have a characteristic appearance and cellular organisation. Regardless of cause, size, or location, mature CNS lesions can be divided into three tissue compartments that each have a unique cell biology: (i) a central non-neural lesion core of stromal cells and fibrotic tissue, (ii) an astrocyte limitans border that surrounds and constrains the stromal cell scar, and (iii) a surrounding zone of viable neural tissue that is spared and functional but reactive and remodelling (Fig. 1). These three compartments exhibit markedly different cellular composition and functional interactions. As described and discussed below, multiple types of loss-of-function technologies are being applied to dissect the roles of different cell types and the molecules that they use to interact during responses to CNS insults that result in scar formation and tissue fibrosis.

## Experimental loss-of-function models to study cells and molecules in vivo

Various genetically targeted procedures are available to achieve cell-type-specific loss-of-function manipulations. These include methods that ablate specific cells and methods that delete specific molecules from specific cells. As with all powerful technologies used in experimental biology, it is important to understand their limitations and potential confounds, and to apply them rigorously and with appropriate controls, and to interpret results cautiously and appropriately.

Selective ablation of specific neural cell types that are proliferating can be achieved by transgenically targeting the expression of herpes simplex virus thymidine kinase (HSVTK) and then administering the antiviral drug ganciclovir (GCV) (Bush et al. 1999, 1998; Garcia et al. 2004). HSVTK (but not mammalian TK) will phosphorylate the GCV into a thymidine analogue that incorporates into DNA. When mammalian cells expressing HSVTK are exposed to GCV, the phosphorylated GCV is trapped and accumulates in the cells, and if the cells attempt to proliferate the DNA that contains GCV disrupts cell division and the cells die via and apoptotic mechanism (Borrelli et al. 1988; Tomicic et al. 2002) that on its own induces minimal, if any, infiltrative inflammation when cells are ablated that do not directly regulate inflammation (Borrelli et al. 1989; Jiang et al. 2011). This TK + GCV procedure has been widely used to study the effects in multiple disorder models of ablating a variety of different types of proliferating cells, including astrocytes (Anderson et al. 2016; Bush et al. 1999; Faulkner et al. 2004; Myer et al. 2006; Voskuhl et al. 2009), microglia (Bennett and Brody 2014; Lalancette-Hebert et al. 2007), OPCs (Hesp et al. 2018), and fibroblasts (Dorrier et al. 2021) (see also Table 1). Cell proliferation can also be attenuated by transgenically targeting molecules that inhibit cell-cycle progression (Dias et al. 2018). Ablation of non-proliferating cells can be achieved by transgenically targeting the expression of diphtheria toxin receptor (DTR) to specific cells and administering ultra-low doses of diphtheria toxin A (DTA) that do not harm other mouse cells (Anderson et al. 2016; Buch et al. 2005). In addition to genetically targeted strategies, certain chemical agents have also been identified that can selectively ablate microglia, such as the molecule PLX3397 and PLX5622 which act by inhibiting the colony stimulating factor 1 receptor (CSF1R) (Bellver-Landete et al. 2019; Elmore et al. 2014). Administering and withdrawing the inhibitor (PLX5622) allows for rapid repopulation of CNS tissue with resident microglial progenitors, thus allowing for transient and timed depletion studies (Elmore et al. 2018; Rice et al. 2017).

**Table 1:** Effects of cellular or molecular loss-of-function manipulations of glial or stromal cells in CNS disorders

Cell type targeted	Loss-of-function model	Disorder model	Loss-of-function consequences for scar formation and neural repair	References
*Astrocytes*	Cell ablation, TK + GCV, DTR	TBI, SCI, EAE	Failure of astrocyte border formation, increased spread of inflammation, increased lesion size, increased loss of neural parenchyma, failure to form a secondary blood–brain barrier, reduced axon regeneration, increased loss of neurological function	Bush et al. (1999), Faulkner et al. (2004), Myer et al. (2006), Voskuhl et al. (2009), Anderson et al. (2016)
*Astrocytes*	*Stat3*-cKO, *Gp130*-cKO, *Notch1*-cKO, *Map3k13*-cKO, *Yap*-cKO	SCI, EAE, infection, stroke	Reduced proliferation, reduced migration, failure of astrocyte border formation, increased spread of inflammation, increased lesion size, increased loss of neural parenchyma, reduced axon regeneration, increased loss of neurological function	Herrmann et al. (2008), Wanner et al. (2013), Drogemuller et al. (2008), Haroon et al. (2011), Shimada et al. (2011), Anderson et al. (2016), Chen et al. (2018), Xie et al. (2020)
*Astrocytes*	*Cldn4*-cKO	EAE	Failure to form a secondary blood–brain barrier after disruption of the endothelial barrier	Horng et al. (2017), Mora et al. (2020)
*Astrocytes*	*ERα*-cKO, *Tgfβ*-cKO	EAE, stroke	Increased inflammation, reduced neurological function	Spence et al. (2011), Cekanaviciute et al. (2014)
*Astrocytes*	*Nfkb*-cKO, *Vgfa*-cKO, *Ccl2*-cKO, *Cxcl10*-cKO	EAE, SCI	Reduced inflammation, increased permeability of endothelial blood–brain barrier	Brambilla et al. (2005, 2009), Argaw et al. (2012), Kim et al. (2014), Ko et al. (2014)
*Astrocytes*	*Gfap* + *Vim*-KO	Stroke	Attenuated of astrocyte border formation, increased spread of inflammation, increased lesion size	Li et al. (2008), Liu et al. (2014)
*Microglia*	Cell ablation, PLX5622, PLX3397	SCI	Increased lesion size, exacerbated degeneration with increase loss of neurons and oligodendrocytes, impaired functional recovery	Bellver-Landete et al. (2019), Fu et al. (2020)
*Microglia*	TK + GCV	Stroke, TBI, SCI	Increased lesion size and greater neuron loss after stroke, no reduction of axon degeneration after TBI	Lalancette-Hebert et al. (2007), Bennett and Brody (2014)
*Microglia*	*Plxnb2*-cKO		Reduced microglial motility, contact inhibition of locomotion, reduced communication with astrocytes, hampered lesion corralling	Zhou et al. (2020)
*Microglia*	*VPS35-*cKO	Stroke	Increased anti-inflammatory microglia, reduced neuronal death, and sensorimotor deficits	Ye et al. (2019)
*Microglia*	*Atg7*-cKO	EAE	Reduced autophagosome formation, reduced debris clearance, impaired myelin degradation, reduced behavioural recovery	Berglund et al. (2020)
*OPCs*	Cell ablation, NG2-TK + GCV	SCI	Impaired astrocyte border formation, reduced stromal cell scar tissue, increased oedema, impaired motor recovery	Hesp et al. (2018)
*OPCs*	Stat3-cKO, Socs3-cKO, *ErbB-cKO*	SCI	Reduced remyelination (no comments recorded regarding potential effects on scar formation)	Hackett et al. (2016), Bartus et al. (2019)
*Stromal cells: Type-A pericytes*	Block proliferation	SCI	Partial reduction of stromal cell scarring derived specifically from Type-A pericytes improved tissue repair, whereas pronounced Type-A pericyte ablation and substantial prevention of stromal scarring worsened outcome	Dias et al. (2018)
*Pericytes*	*Postn*-KO	SCI	Suppressed pericyte proliferation, reduced expression of TNFα from myeloid cells, decreased scar formation, slower functional recovery	Yokota et al. (2017)
*Stromal cells: perivascular fibroblasts*	Cell ablation	EAE	Reduced ECM deposition and tissue fibrosis, increased migration of OPCs into lesion areas	Dorrier et al. (2021)

Deletion of specific molecules from specific cells can be achieved in several ways. The bacteriophage Cre-recombinase-loxP system is the basis for several approaches. Genes encoding molecules of interest are targeted by insertion of loxP sites into regions of the gene essential for expression. Cell-type specificity is achieved by transgenic targeting of the Cre enzyme using cell-type-specific promoters. In cells that express the Cre enzyme, the loxP sites are recombined, thereby excising the essential gene sequences and stopping expression of the molecule (Nagy 2000). When used with loxP-STOP-loxP transgenes, Cre-loxP technology can also be used to express molecules such as reporter molecules or functionally active molecules in specific cell types. Basic Cre-loxP technology results in constitutive gene deletion starting with the first expression of the promoter during development. In addition, there are now temporally controllable methods in which the onset and duration of Cre expression can be controlled. The most commonly used of these methods at present is a Cre modified to be controlled by an engineered estrogen receptor that responds to the presence of the drug tamoxifen (Cre-ERT). This inducible Cre-ERT is also transgenically targeted to specific cell types, but is silent until tamoxifen is administered. Cre-ERT is active only as long as tamoxifen is present, which allows the targeting of gene deletions to mature cells and avoid developmental effects of molecular deletions. Temporally restricted Cre-ERT to express reporter molecules can also be used for the cell lineage mapping of progeny derived from proliferating cells. A caveat associated with the use of tamoxifen-inducible Cre technology in experimental models of CNS injury and disease, is that tamoxifen has potent anti-estrogen properties and that estrogen signalling can be important in certain CNS responses to injury and disease. It is therefore essential to allow sufficient time for tamoxifen to be cleared after induction of Cre and administer tamoxifen to control subjects. Constitutive and inducible Cre-loxP technology has been used extensively to dissect the roles of specific molecules derived from specific cell types in the multiple CNS disorder models that involve inflammation and scar formation (Table 1). Attenuation of molecular expression using short inhibitory RNAs (siRNAs) (Zheng et al. 2018) is also being explored in CNS injury models with inflammation and scar formation (Al Mamun et al. 2020).

## Loss-of-function studies: astrocytes

Astrocytes are CNS neural parenchymal cells that derive from the same neural stem and progenitor cells as neurons and oligodendrocytes and are of neural ectoderm origin (Molofsky and Deneen 2015). In healthy CNS tissue, astrocytes exert many activities essential for normal neurological function: they maintain homeostasis of extracellular fluids, ions, and transmitters; provide glucose metabolites as energy substrates to neurons; modulate local blood flow; play essential roles in synapse development and plasticity; and exhibit dynamic activities crucial for neural circuit function, sensory-motor function, cognition, and behaviour, as reviewed elsewhere (Khakh 2019). In response to injury and disease, astrocytes become reactive and can exhibit a wide range of responses ranging from subtle changes in gene expression to proliferation and formation of borders around scar tissue formed by stromal cells (Sofroniew 2020; Sofroniew and Vinters 2010). Astrocytes also form borders around foreign bodies such as biomaterials implanted for therapeutic purposes (O’Shea et al. 2020). Roles of reactive astrocytes and their contributions to scar formation in diverse CNS disorders have been studied extensively with a wide variety of causation-testing genetically targeted loss-of-function experimental models involving either cell ablation or selective deletion of specific molecules (Table 1). Early studies using TK + GCV to ablate proliferating astrocytes provided the first evidence that astrocyte borders around stromal cell scars constrained the spread of infiltrating inflammatory cells and thereby protected adjacent neural tissue and preserved function (Bush et al. 1999; Faulkner et al. 2004). Notably, the increased and prolonged spread of infiltrative inflammation observed after ablation of astrocytes in CNS injury models is not an artefact of the ablation technology, but has experimentally been demonstrated to result from a loss of astrocyte regulation and restriction of infiltrating immune cells; such inflammation does not occur when neurons and oligodendrocytes are ablated, and similar inflammation occurs when certain molecules are deleted selectively from astrocytes in the absence of cell ablation (Faulkner et al. 2004; Herrmann et al. 2008; Sofroniew 2015; Wanner et al. 2013). Astrocyte-targeted deletion of a large variety of different molecules by multiple different laboratories has now shown that astrocytes use specific molecular signalling mechanisms to help attract circulating immune and inflammatory cells to CNS lesions to clear debris, as well as to corral these cells within lesion areas and prevent their spread into adjacent viable neural tissue (Table 1). A large literature on this topic has been reviewed elsewhere (Sofroniew 2015, 2020). Key molecules used by astrocytes in the regulation of CNS inflammation and scar formation include Nfkb, Vegfa, Ccl2, and Cxcl10 that help to attract peripheral immune and inflammatory cells to help clear infections or necrotic debris; Tgfβ and ERα that exert anti-inflammatory functions; Cldn4 that forms astrocyte tight junctions to establish a secondary blood–brain barrier in CNS parenchyma beyond the endothelial barrier; and Stat3, Notch1, Map3k13, and Yap that help coordinate intracellular signalling involved in astrocyte proliferation and the formation of astrocyte borders that constrain the spread of peripheral immune and inflammatory cells (see Table 1). Although historically there has been a long-standing view of astrocyte responses as being detrimental to injury and disease, loss-of-function studies have for the most part identified essential beneficial and tissue reparative effects of astrocyte responses. Even the longstanding view that reactive astrocytes are the primary cause for the failure CNS axon regeneration has been overturned. Preventing astrocyte border formation by ablating proliferating astrocytes with GFAP-TK, or by ablating chronic astrocyte borders using DTR, or by attenuating border formation through deletion of Stat3, all fail to result in spontaneous CNS axon regeneration and attenuate stimulated axon regrowth (Anderson et al. 2016, 2018; Sofroniew 2018). In addition, recent gain-of-function molecular manipulations report that increasing astrocyte proliferation and border formation, for example by increasing the activity of Map3k13 or Yap, can improve repair after SCI (Chen et al. 2018; Xie et al. 2020).

## Loss-of-function studies: microglia

Microglia are CNS resident cells that exert many activities essential for neurological functions in the healthy CNS and in response to CNS disorders (Prinz et al. 2019; Wolf et al. 2017). Although microglia are integral components of CNS neural functions, they are not of neural ectoderm origin. Microglia derive from the yolk sac and populate CNS during early embryonic development (Ginhoux et al. 2010) and replenish throughout life by proliferation within the CNS (Askew et al. 2017). In the healthy CNS, essential functions of microglia include synaptic remodelling (Schafer et al. 2012), neuroprotection (Chen et al. 2014), modulation of neurogenic niches (Sierra et al. 2010), contribution to myelin turnover and remyelination (Lloyd and Miron 2019), and contribution to blood–brain barrier maintenance (Haruwaka et al. 2019). In response to CNS disorders, essential functions of microglia include enhanced phagocytosis (Berglund et al. 2020), enhanced synaptic pruning (Yoshiyama et al. 2007), self-proliferation, and release of cytokines and chemokines to activate and recruit other cell types into CNS inflammatory responses (Prinz et al. 2019; Wolf et al. 2017). Roles of microglia in diverse CNS disorders have been studied with a variety of loss-of-function experimental models involving either cell ablation or selective deletion of specific molecules that have identified numerous beneficial and tissue reparative effects of microglial responses (Table 1). For example, selective ablation of microglia using the Plexxicon molecules PLX3397 or PLX5622, which act by inhibiting microglia CSF1R, markedly exacerbated tissue loss and impaired functional recovery after SCI (Bellver-Landete et al. 2019; Fu et al. 2020). It should be noted that these Plexxicon compounds also affect CSF1R expressing macrophages (Lei et al. 2020) and are currently in clinical trials for targeting tumour-associated macrophages (Wesolowski et al. 2019), and this needs to be taken into account when evaluating observations in experimental models of CNS injury or disease that involve compromise of the blood–brain barrier (BBB) and infiltration of blood-borne macrophages. A new CSF1R inhibitor, PLX73086, does not deplete CNS resident microglia due to low penetration across the BBB, and may thus help delineate myeloid cell roles (Bellver-Landete et al. 2019; Nestor et al. 2018). Microglial ablation models have been used in a variety of other experimental contexts. For example, microglial ablation does not prevent the retrograde death of axotomised retinal neurons (Hilla et al. 2017) contrary to suggestions that microglia induce retinal astrocytes to kill retinal neurons that have been axotomised. Interestingly, PLX3397 ablation of microglia showed that microglia are essential for and coordinate the ‘scar-free’ healing and axon regeneration that occurs after SCI in neonatal mice, and that neonatal scar-free healing may be related to peptidase inhibitors expressed by neonatal (but not adult) microglia (Li et al. 2020). Various studies have selectively deleted specific molecules from microglia. In adult mice, the tamoxifen-regulated deletion of *Plxnb2* selectively from Cx3cr1-expressing microglia showed that these cells play critical roles in the corralling and restriction of CNS inflammatory lesions, and that without microglia there is increased spread of inflammation, increased tissue damage, and reduced recovery after SCI (Zhou et al. 2020). HDAC3 inhibition preferentially modulated gene expression in microglia that are proliferating after SCI, and enhanced wound repair and suppressed inflammatory cytokine release in an acute SCI setting (Wahane et al. 2021). Other studies show that mice in which *Atg7* has been selectively deleted from microglia exhibit slower recovery from EAE, less autophagosome formation, reduced debris clearance, and impaired myelin degradation (Berglund et al. 2020).

## Loss-of-function studies: OPCs

OPCs are CNS neural parenchymal cells that are tissue resident progenitor cells that derive from the same neural stem and progenitor cells as neurons and astrocytes and are of neural ectoderm origin. OPCs are best known for generating new oligodendrocytes throughout life in both health and disease (Duncan et al. 2020; Levine et al. 2001). In addition, it is now clear that OPCs are multipotent progenitors that can also differentiate into astrocytes in certain regions of healthy CNS and under certain injury or inflammatory conditions (Hackett et al. 2016, 2018; Nishiyama et al. 2016). Targeting OPCs for transgenic manipulations is complicated because they share molecular markers with other cells. OPCs express the molecule NG2 (CSPG4) and are sometimes referred to as NG2-glia or NG2-OPCs (Nishiyama et al. 2016). Although NG2 has been used to target OPCs for genetic manipulations in experimental models, it is important to note that NG2 (CSPG4) is also expressed by pericytes (Vanlandewijck et al. 2018), which are perivascular stromal cells that can give rise to pathological fibroblasts, and this diverse expression complicates the interpretation of results using NG2-targeted transgenic manipulations in CNS disorder models that include stromal cell scar formation. Another lineage marker that can be used to target OPCs is Pdgfra (Nishiyama et al. 1996), but this too can be expressed by other cell types including perivascular stromal cells (Vanlandewijck et al. 2018), so caution is also needed in interpreting results of this transgenic manipulation in studies of OPCs in disorder models.

Until recently, studies on OPCs in disorder models have focused exclusively on their generation of remyelinating oligodendrocytes. Nevertheless, there is growing interest in examining the potential contribution of OPCs to the glial cell borders that form around stromal cell scars. Potential contributions could be either directly whilst retaining features of OPCs in their own right, or indirectly by OPCs giving rise to progeny cells that have the features of astrocytes. Transgenically targeted cell lineage and fate mapping studies with NG2 and Pdgfra have shown that OPCs can generate newly proliferated progeny cells that express astrocyte markers such as Gfap and Aldh1l1, which intermingle with astrocytes in borders around stromal cell scar tissue after SCI (Hackett et al. 2018). The role of these OPC-derived astrocyte-like cells in the borders is not yet clear. Only a few loss-of-function studies have to date tried to examine OPC roles in this capacity. One study used TK + GCV to ablate proliferating NG2-expressing cells after SCI, which targeted both OPC and pericytes. This ablation resulted in discontinuous astrocyte borders around stromal cell scar tissue as well markedly reduced stromal cell scar tissue, with resultant increased oedema, prolonged haemorrhage, and impaired motor recovery (Hesp et al. 2018). Because NG2 ablation targets both OPC and pericytes it is not yet clear which cell type contributes more to these observations. No studies appear to date to have examined the effects on the formation of glial borders around stromal cell scars of specific molecular deletions from OPCs. One molecular deletion study shows that removal of ErbB receptor from OPC targeted via Pdgrfa-Cre reduces their formation of remyelinating Schwan cells and attenuates remyelination after SCI (Bartus et al. 2019), and another shows that deletion of Stat3 or Socs3 from OPC via NG2-Cre reduced remyelination after SCI (Hackett et al. 2016), but neither study reported any effects on lesion size or astroglial borders.

## Loss-of-function studies: stromal cells

In the CNS, as in other organs throughout the body, true scar tissue is formed by the expansion of perivascular stromal (mesenchymal) cells and the fibrotic extracellular matrix (ECM) that they produce (Di Carlo and Peduto 2018). In healthy CNS tissue, perivascular stromal cells are essential components of the vasculature. In response to injury and disease, the perivascular niche in the CNS, as in other organs, contains different stromal cell populations that can rapidly generate pathological fibroblasts that deposit fibrotic ECM, including perivascular fibroblasts, pericytes, and mesenchymal stem cells (Di Carlo and Peduto 2018). This rapid proliferation of pathological fibroblasts occurs to quickly replace lost or damaged parenchymal cells so as to preserve tissue architecture and organ function until new parenchymal cells can be generated when possible. Long-term scaring occurs when parenchymal replacement is incomplete or poor. In the CNS, cell-lineage tracing studies using transgenic markers have identified Type-A pericytes (Goritz et al. 2011) and collagen-producing perivascular fibroblasts (Dorrier et al. 2021; Soderblom et al. 2013) as sources of pathological fibroblasts that form stromal cell scar tissue and deposit fibrotic ECM in the CNS after SCI. The effects of selective cell ablation studies have been performed on both of these cell populations in different disorder models. After SCI, a partial reduction of Type-A pericyte-derived stromal cell scarring, achieved by transgenic attenuation of pericyte proliferation, appears to improve tissue repair and promote locomotor recovery, whereas a pronounced Type-A pericyte ablation and a more complete prevention of the scarring produced by them appears to be detrimental and worsen outcome (Dias et al. 2018). In the CNS inflammation model of autoimmune encephalomyelitis (EAE), TK + GCV-mediated ablation of proliferative collagen-producing perivascular fibroblasts significantly reduced ECM deposition and tissue fibrosis, which resulted in increased migration of OPCs into lesion areas (Dorrier et al. 2021). These findings support long-standing notions that modulating stromal cell scar formation and its associated ECM deposition and tissue fibrosis represent potential targets in various CNS disorders. Notably, in the EAE model, no evidence was found for a contribution of pericytes to fibrotic scar formation or tissue fibrosis (Dorrier et al. 2021). In addition, conditional deletion of IFNgR1 in fibroblasts after EAE reduced fibrotic scar formation and tissue fibrosis (Dorrier et al. 2021). To date, there do not appear to be any other specific molecular deletion studies targeted to pathological fibroblasts that form stromal cell scars in models of CNS disorders. However, such studies in other organs show for example that deletion of interleukin1-receptor1 (IL1R1) from pathological fibroblasts reduces cardiac fibrosis and improved cardiac function after experimental myocardial infarction (Bageghni et al. 2019). This type of experimentation warrants further study in models of CNS disorders.

## Multicellular interactions contribute to CNS scar formation

The response to CNS tissue damage or degeneration at all scales from large lesions to small local perturbations involves complex interactions of multiple types of CNS glia and non-neural cells that can lead to scar formation and tissue fibrosis. The loss-of-function studies examined and discussed above show that different types of CNS glial cells play essential roles in the repair of CNS tissue damage or degeneration, in particular by interacting to surround and corral stromal cell scars and fibrosis. Notably, attenuating certain specific functions of CNS resident glial cells, in particular astrocytes and microglia, and potentially OPCs, can disrupt the formation of the astrocyte limitans borders that form around stromal cell scars and lead to increased spread of excess inflammation, increased loss of neural tissue, and increased fibrosis. These observations are beginning to provide insights into specific neuropathological mechanisms whereby specific dysfunctions of different types of CNS glia caused by disorder-related mechanisms, such as genetic defects, genetic polymorphisms, or disease-related perturbations in molecular signalling, could either precipitate or contribute to disorder-related neurological dysfunction and tissue loss or fibrosis. Thus, CNS glia, as well as fibrosis-producing stromal cells, can now be considered as potential major contributors to diverse CNS disorders either through loss- or gain-of-functions, and are thereby emerging as important potential targets for interventions. Nevertheless, our knowledge base is still small and the molecular signals that control glial cell interaction amongst themselves and with stromal cells are only beginning to be elucidated. Dissecting such mechanisms and understanding why they occur and how they can be targeted for beneficial manipulation, has the potential to help develop novel approaches to improving outcomes in multiple CNS disorders.

## Similarities amongst CNS scars, limitans borders, and secondary lymphoid follicles

As discussed above, CNS scar formation can occur across a wide range of scales from large lesions to small tissue perturbations. Notably across all of these scales, scar formation exhibits a similar cytoarchitectural organisation consisting of central cores of scar-forming stromal cells with fibrotic matrix and infiltrating peripheral inflammatory cells that are surrounded by limitans borders formed by astrocytes and other CNS resident glia (Fig. 1) (Burda and Sofroniew 2014). It is also noteworthy that this general structure of CNS lesions and scars is similar across responses to many forms of CNS insults in which there is tissue damage, such as traumatic injury, ischemic stroke, infection, autoimmune infiltration, neurodegenerative processes, and even the response to foreign bodies such as implanted biomaterials (O’Shea et al. 2020; Sofroniew 2020; Sofroniew and Vinters 2010).

In trying to dissect mechanisms of CNS scar formation and understand why they occur and how to generate therapeutic manipulations that might improve outcomes, it can be instructive to compare the very generalised cytoarchitectural organisation of CNS lesions with the mechanisms and functions of other similarly appearing CNS cytoarchitectural assemblies. In the CNS, as in other organs, scar formation occurs when parenchymal cells die or degenerate. Parenchymal cell death and tissue damage trigger innate immune responses to clear debris and monitor for potential infection, which in the CNS gives rise to two opposing needs, (i) to recruit peripheral immune and inflammatory cells and (ii) to separate non-neural stromal and inflammatory cells from functioning CNS neural parenchyma. Notably, CNS cytoarchitectural assemblies that exert both of these functions exist in contexts that are not considered scar formation.

Along the entire surface of the healthy CNS, an astrocyte limitans border separates functioning CNS neural parenchyma from the adjacent stromal cells of the meninges that form protective layers under continuous immune surveillance. Similarities between the astrocyte borders along the stromal cells of the meninges in healthy CNS and the astrocyte borders around stromal cell scars in CNS lesions have been noted at cytoarchitectural, molecular, and functional levels, and disruption of both types of borders leads to the spread of destructive inflammation into CNS parenchyma and neural tissue loss (Sofroniew 2015). Formation of astrocyte borders along meninges occurs during development, and it will be interesting to study whether border formation around stromal cell scar formation recapitulates any of the developmental genetic programs.

Regarding the recruitment of immune and inflammatory cells, there is now growing interest in the formation and maintenance of adventitial (perivascular) cuffs or secondary lymphoid follicles within host tissues as important aspects of immune responses across multiple organs (Dahlgren and Molofsky 2019). CNS secondary lymphoid follicles are cytoarchitecturally similar to CNS scars, with central cores of immune cells intermingled with stromal cells and extracellular matrix. In the CNS, the formation of secondary lymphoid follicles and local antibody production are emerging as fundamental mechanisms to clear viral infections (Cupovic et al. 2016; Martin and Griffin 2018; Metcalf et al. 2013). The formation of secondary lymphoid follicles during viral infections depends upon stromal cell (fibroblast) expansion and extracellular matrix deposition to form essential scaffolds that both facilitate and help to control immune cell infiltration (Cupovic et al. 2016). CNS secondary lymphoid follicles surrounded by astrocyte borders also form during autoimmune disorders where border-forming astrocytes take part both in attracting and constraining the recruited immune and inflammatory cells as discussed above and in Table 1. The mechanisms that facilitate the clearing of viral infections are likely to be evolutionarily ancient and have influenced the evolution of cellular and molecular mechanisms generated in response to other CNS insults. It will be interesting to compare the molecular mechanisms that drive the formation of secondary lymphoid follicles in response to viral infections with the mechanisms that drive the recruitment and corralling of immune and inflammatory cells during CNS scar formation triggered by tissue loss in diverse disorders such as trauma, ischemia, neurodegenerative disease, and other CNS insults.

## It is time to retire the term ‘glia scar’

In all organs throughout the body, scar tissue is defined as the replacement of host organ parenchymal cells by stromal (mesenchymal) cells and fibrotic extracellular matrix (Rhett et al. 2008; Rog-Zielinska et al. 2016). True scar tissue consists of non-neural stromal cells and fibrotic ECM, and does not contain functioning tissue and will not spontaneously repair or contribute to functional recovery. By this definition, CNS glial cells do not form scar tissue. Glia are CNS parenchymal cells that proliferate in response to injury or disease in order to promote tissue repair by replacing lost neural parenchyma with new neural parenchyma rather than with fibrotic tissue. Only where glial cells are present will neuronal elements survive and be able to contribute to restoration of neurological functions.

In no other organ is the proliferation of essential parenchymal cells regarded as scar formation. Yet in the CNS, the term ‘glial scar’ is in common usage. The term ‘glia scar’ was first coined by nineteenth-century anatomists who regarded glia as connective tissue elements and who were not aware of the neural origin or essential contributions of glia to neural function. The use of the term ‘glia scar’ has been passed on from generation to generation of researchers over many decades largely without reflection of its implications and without mechanistic assessment of its validity. Over a hundred years later, on the basis of a large volume of recent work it is appropriate to make use of advances in knowledge showing that astrocytes and OPCs are CNS resident glia that derive from the same neural progenitors as neurons (Gage 2000) and are true neural parenchyma that continually perform activities essential for neurological functions. As with parenchymal cells in other organs, CNS glia proliferate after injury or disease in order to replace lost parenchyma and to protect adjacent surviving neural parenchyma. They are not mesenchymal or stromal cells and they do not contribute to stromal cell scars or its associated fibrosis. Experts in human CNS pathology and astrocyte pathophysiology have previously advocated that astrocytes responding to tissue damage should not be confused with true mesenchymal scar and have questioned the practice of referring to astrocyte responses as ‘scars’ (Norenberg et al. 2004). As discussed in this article and elsewhere (Sofroniew 2020), steadily mounting experimental evidence supports this view. Loss-of-function studies, as reviewed above, have revealed the essential tissue reparative functions of glial that proliferate to replace CNS parenchyma lost to injury or disease. Based on this evidence, we advocate that it is time to put the term ‘glial scar’ to rest and restrict the term ‘scar’ to non-neural stromal cells and fibrotic extracellular matrix in line with terminology in other tissues. Use of the term ‘glial scar’ has fostered a wide-spread negative view in which students or new investigators entering the field become biased that glial responses to injury are detrimental in the first instance and therefore require attenuation. This is not the case and it is time to move on from this terminology.

What then should glial cells that proliferate around stromal cell scars and function to corral and constrain them be referred to as? A simple but accurate term would be ‘border-forming’ glia that proliferate in response to injury, disease, and inflammation. These new borders are similar in appearance and function to the astrocyte limitans borders along meninges that line the entire external margins of the healthy CNS. In both cases the astrocyte borders serve to delineate neural from non-neural tissue. This similarity has long been recognised and this term and concept of limitans border forming glia that surround and constrain or corral stromal cells and infiltrating immune and inflammatory cells has been in consideration and use for some time and has both structural and functional accuracy (Sofroniew 2020).

## Knowledge gaps and future directions

Loss-of-function studies have begun to reveal a variety of important roles played by different neural and non-neural cell types in CNS inflammation, scar formation, and fibrosis in response to different CNS insults. Nevertheless, our understanding is still limited and more work is needed in particular in dissecting the complex and multifactorial molecular mechanisms that regulate and influence the balance between tissue loss or tissue repair. This type of information is necessary to begin developing evidence-based strategies for beneficial interventions. Notably in this regard, and somewhat contrary to past expectations, most loss-of-function manipulations of CNS glial cells have resulted in detrimental effects, highlighting the importance of these cells in multiple aspects of repair. These observations suggest that rather than attempting to block effects of glial cells, it may be more appropriate to augment certain of their functions and effects. Thus, as information accrues, use of both loss-of-function and gain-of-function experimental manipulations may be useful for testing the impact of targeting specific mechanisms for their potential beneficial effects and their potential unwanted side effects in specific disorder contexts. In addition, tissue fibrosis due to stromal cell deposition of ECM in response to chronic low-grade inflammation is increasingly recognised as a major cause of dysfunction in organs such as heart, lungs, and kidney, but has received little attention in CNS disorders. It deserves more extensive investigation in multiple contexts, including chronic accumulation of low-grade fibrosis.
