# Correction to: Loss‑of‑function manipulations to identify roles of diverse glia and stromal cells during CNS scar formation

**DOI:** 10.1007/s00441-022-03617-w

**Published:** 2022-04-02

**Authors:** Shalaka Wahane, Michael V. Sofroniew

**Affiliations:** Department of Neurobiology, David Geffen School of Medicine, University of California, Los Angeles, CA 90095 USA

## Correction to: Cell and Tissue Research https://doi.org/10.1007/s00441-021-03487-8

The authors regret that the title of the article that appears in the original article is incorrect. The correct article title is “Loss‑of‑function manipulations to identify roles of diverse glia and stromal cells during CNS scar formation” which is now captured in this erratum article.

The original article has been corrected.

